# Multiplex One-Step RT-qPCR Assays for Simultaneous Detection of SARS-CoV-2 and Other Enteric Viruses of Dogs and Cats

**DOI:** 10.3390/v15091890

**Published:** 2023-09-07

**Authors:** Côme J. Thieulent, Mariano Carossino, Laura Peak, Wendy Wolfson, Udeni B. R. Balasuriya

**Affiliations:** 1 Louisiana Animal Disease Diagnostic Laboratory, School of Veterinary Medicine, Louisiana State University, Baton Rouge, LA 70803, USA; cthieulent@lsu.edu (C.J.T.); mcarossino1@lsu.edu (M.C.); lpeak1@lsu.edu (L.P.); 2 Department of Pathobiological Sciences, School of Veterinary Medicine, Louisiana State University, Baton Rouge, LA 70803, USA; 3 Department of Veterinary Clinical Sciences, School of Veterinary Medicine, Louisiana State University, Baton Rouge, LA 70803, USA; wwolson@lsu.edu

**Keywords:** reverse transcription–qPCR (RT-qPCR), multiplex, feline enteric virus, canine enteric virus, SARS-CoV-2, diagnostics, zoonosis, reverse zoonosis

## Abstract

The severe acute respiratory syndrome coronavirus-2 (SARS-CoV-2) was transmitted from humans to dogs and cats (reverse zoonosis) during the COVID-19 pandemic. SARS-CoV-2 has been detected in fecal samples of infected dogs and cats, indicating potential fecal–oral transmission, environmental contamination, and zoonotic transmission (i.e., spillback). Additionally, gastrointestinal viral infections are prevalent in dogs and cats. In this study, we developed and validated a panel of multiplex one-step reverse transcription–quantitative polymerase chain reaction (RT-qPCR) assays for the simultaneous detection of SARS-CoV-2 and common canine enteric viruses: Canine Enteric Assay_1 (CEA_1) for the detection of canine adenovirus-1, canine enteric coronavirus, canine distemper virus, and canine parvovirus, and CEA_2 for the detection of rotavirus A (RVA), and SARS-CoV-2); or common feline enteric viruses (Feline Enteric Assay_1 (FEA_1) for the detection of feline enteric coronavirus, feline panleukopenia virus, RVA, and SARS-CoV-2). All assays demonstrated high analytical sensitivity, detecting as few as 5–35 genome copies/µL in multiplex format. The repeatability and reproducibility of the multiplex assays were excellent, with coefficient of variation <4%. Among the 58 clinical samples tested, 34.5% were positive for at least one of these viruses, and SARS-CoV-2 was detected in two samples collected from one dog and one cat, respectively. In conclusion, these newly developed one-step multiplex RT-qPCR assays allow for rapid diagnosis of enteric viral infections, including SARS-CoV-2, in dogs and cats.

## 1. Introduction

The COVID-19 pandemic, caused by severe acute respiratory syndrome coronavirus 2 (SARS-CoV-2) [1,2], had a significant impact on human health and the global economy. While human-to-human transmission remains the primary route of infection, interspecies transmission has been demonstrated, including to companion animals [3,4]. Among those, dogs and cats, which share close contact with humans, have received particular attention as potential sources of SARS-CoV-2 infection following transmission from humans [5,6]. In addition to human respiratory secretions, SARS-CoV-2 has been detected in various biological samples, including feces, suggesting the potential for viral shedding through the gastrointestinal tract [7,8,9,10]. The detection of viral RNA in the feces of infected humans has prompted the development of surveillance systems by testing wastewater, as well as investigations into the possibility of fecal–oral transmission [11,12,13,14]. Several studies have also reported the detection of SARS-CoV-2 RNA in the feces and rectal swabs collected from naturally infected dogs [15,16,17,18] and cats [19,20,21,22]. Furthermore, Gaudreault et al. [23] demonstrated viral RNA presence in the fecal samples of experimentally infected cats, highlighting the potential for viral dissemination via fecal shedding. Additionally, viral RNA was detected in rectal swabs collected from experimentally infected dogs [24]. These observations raise important questions regarding the infectivity of SARS-CoV-2 shed in feces, and the duration of viral excretion in companion animals.

Besides SARS-CoV-2, gastrointestinal diseases are frequently reported in companion animals [25]. Bacterial, parasitic, and viral agents can all contribute to the development of enteric illnesses in dogs and cats, leading to high mortality rates in kittens, puppies, and unprotected populations [26,27]. Canine parvovirus (CPV) and canine enteric coronavirus (CECoV) are widely recognized as the most prevalent enteric pathogens worldwide [28,29]. Additionally, canine distemper virus (CDV) is commonly implicated in enteric disorders [30,31]. Canine adenovirus type 1 (CAdV-1) is typically associated with infectious canine hepatitis, but it can also cause severe gastrointestinal disease with shedding through feces and urine [32]. Feline enteric coronavirus (FCoV) [33,34] and feline panleukopenia virus (FPV) [35,36] are the two most important viral causes of feline gastrointestinal disease worldwide. Although canine and feline rotavirus A (RVA) are not commonly found as enteric pathogens and rarely cause severe illness, their zoonotic potential makes them significant [37,38,39].

The conventional diagnostic methods for detecting viral infections often involve separate testing procedures, such as virus isolation, classical PCR/reverse transcription (RT)-PCR and quantitative PCR (qPCR)/RT-qPCR, which are laborious and expensive, requiring multiple tests to be performed for each individual infectious agent of interest. To overcome these limitations, multiplex qPCR/RT-qPCR assays have gained popularity due to their ability to simultaneously detect multiple viral DNA and RNA agents in a single reaction. This approach offers several advantages, including reduced turnaround time, improved cost-effectiveness, and enhanced diagnostic accuracy [40]. This technique is now widely used in diagnostic laboratories for the detection of human and animal enteric viruses [41,42,43,44]. Recently, our team developed panels of multiplex qPCR/RT-qPCR for the simultaneous detection of SARS-CoV-2 and pathogens associated with respiratory disorders in cats and dogs [45,46], as well as a quadruplex real-time RT-PCR assay to detect equine rotavirus in diarrheic foals [47].

Testing for SARS-CoV-2 and other cross-species-transmissible viruses (e.g., RVA) in dog and cat fecal samples, coupled with the search for gastrointestinal diseases associated viruses in these species is crucial for proper diagnosis, evaluating zoonotic transmission, assessing environmental contamination, and embracing the One Health approach. Thus, this article describes the development and the validation of a panel of two one-step multiplex RT-qPCR assays for the simultaneous detection and differentiation of canine enteric viruses along with SARS-CoV-2 (Canine Enteric Assay_1 (CEA_1) for the detection of CAdV-1, CECoV, CDV and CPV, and CEA_2 for the detection of RVA and SARS-CoV-2), and a one-step multiplex RT-qPCR assay for the simultaneous detection and differentiation of feline enteric viruses along with SARS-CoV-2 (Feline Enteric Assay_1 (FEA_1) for the detection of FCoV/FIPV, FPV, RVA and SARS-CoV-2). These new assays were used to test clinical specimens submitted to the Louisiana Animal Disease Diagnostic Laboratory (LADDL) from dogs and cats in Louisiana, USA, between 2020 and 2023. The multiplex RT-qPCR assays developed here present a valuable tool for rapid diagnosis, efficient surveillance, and effective disease management of enteric viruses in companion animals. By combining the detection of these viruses in a single reaction, veterinarians, researchers, and public health professionals can obtain comprehensive information on the presence and distribution of these viruses.

## 2. Materials and Methods

### 2.1. Viruses and Bacteria

The panel of reference viruses used for evaluating the specificity (inclusivity/exclusivity) of each RT-qPCR assay in singleplex and in multiplex format is presented in Table 1. The FCoV L1911652 RNA sample was obtained from a formalin-fixed paraffin-embedded section of colon from a deceased cat submitted to the Louisiana Animal Disease Diagnostic Laboratory (LADDL) for routine postmortem examination and confirmed positive for FCoV antigen and negative for FPV antigen via indirect immunofluorescence. Total DNA/RNA was obtained from ten serial 5-micron sections using the QIAamp DNA FFPE Tissue Kit (QIAGEN, Germantown, TN, USA). Canine respiratory coronavirus VSL-1471 and canine influenza A H3N2 VSL-1355 nucleic acids were kindly provided by Dr. Diego Diel (Cornell University). Canine influenza A H3N8 A/Ca/FL/15592/04 nucleic acids were kindly provided by Dr. Edward Dubovi (Cornell University). All other prototype strains were obtained from the American Type Culture Collection (ATCC^®^; Manassas, VA, USA), or BEI Resources (Manassas, VA, USA).

### 2.2. Clinical Specimens

A total of 58 rectal swabs collected from 27 dogs and 31 cats with clinical signs of enteric disease submitted for routine diagnostic testing at the LADDL between 2020 and 2023 were used in this study. The specimens were submitted by practicing veterinarians or the attending veterinarian from the LSU School of Veterinary Medicine Shelter Medicine Program. Rectal specimens were collected using sterile oropharyngeal/nasal swabs (VMRD, Pullman, WA, USA) and resuspended in either 2 mL of BHI Broth (Hardy Diagnostics, Santa Maria, CA, USA) or 2 mL of PrimeStore^®^ molecular transport medium (VMRD). Swab samples were vortexed and clarified with centrifugation at 500× *g* for 5 min at 4 °C.

### 2.3. Nucleic Acid Extraction

Nucleic acids were extracted using the taco^TM^ mini nucleic acid automatic extraction system (GeneReach, Taichung, Taiwan) following the manufacturer’s recommendations. One hundred microliters of rectal swab suspensions were extracted and eluted in the same volume of elution buffer. The extracted nucleic acid samples were stored at −80 °C until used.

### 2.4. Multiplex TaqMan^®^ Reverse Transcription qPCR (RT-qPCR) Assays for the Detection of Canine and Feline Enteric Viruses

Three multiplex one-step RT-qPCR assays were developed and designated as canine enteric assay 1 (CEA_1; targeting CAdV-1, CECoV, CDV, and CPV), CEA_2 (targeting RVA, and SARS-CoV-2) and feline enteric assay 1 (FEA_1; targeting FcoV, FPV, RVA, and SARS-CoV-2). All assays were performed in a total volume of 25 μL containing 12.5 μL of 2X QuantiTect^TM^ Multiplex RT-PCR Master Mix (Qiagen, Hilden, Germany), 0.25 μL of QuantiTect^TM^ RT Mix, 1.25 μL of primers (200 nM) and fluorogenic probes (200 nM) mix, 6 μL of Rnase-free water and 5 μL of template DNA/RNA. All reactions were run on a 7500 Fast Real-Time PCR System (Applied Biosystems, Waltham, MA, USA) with the following thermal profile: a reverse transcription step (20 min at 50 °C) followed by an initial activation step (15 min of at 95 °C) and 40 cycles of denaturation and annealing/extension (45 s at 94 °C and 75 s at 60 °C). All primers and probes for the detection of canine and feline enteric viruses were adopted from previous studies (Table 2). The RVA NSP3-specific (gene segment 7; pan-rotavirus A) assay was established as previously described by Freeman et al. [48] with the addition of a nucleotide degeneracy in position 2 of the NVP3-Fdeg primer (C→Y) based on in silico analysis of the canine and feline RVA NSP3 sequences (available on GenBank https://www.ncbi.nlm.nih.gov/genbank/, accessed on 16 May 2022; Figure S1). Sequences specificities were first verified in silico using NCBI Basic Local Alignment Search Tool (BLAST; https://blast.ncbi.nlm.nih.gov/Blast.cgi?PROGRAM=blastn&PAGE_TYPE=BlastSearch&LINK_LOC=blasthome, accessed on 16 May 2022) and self-annealing sites, hairpin loop formation, and 3′ complementarity were evaluated using IDT’s OligoAnalyzer tool (https://www.idtdna.com/calc/analyzer, accessed on 16 May 2022). Primers and probes were synthesized using Invitrogen (Waltham, MA, USA) and the specificity was evaluated using the panel of prototype strains described above. The complete step-by-step protocols for the detection of both canine and feline enteric viruses have been deposited on https://protocols.io, accessed on 25 July 2023, with the following DOIs: dx.doi.org/10.17504/protocols.io.e6nvwd5z9lmk/v1 and dx.doi.org/10.17504/protocols.io.3byl4qk5jvo5/v1, respectively.

### 2.5. Synthesis of In Vitro Transcribed RNA

Target-specific in vitro transcribed (*IVT*) RNA was synthesized in order to determine the analytical sensitivity of each multiplex RT-qPCR assay as previously described [45,46,47]. Inserts containing the target regions of each assay (flanked by PstI and HindIII restriction enzymes) were chemically synthesized and cloned into the multiple cloning site of the pGEM^®^-3Z vector (Promega, Madison, WI, USA).

### 2.6. Analytical Parameters Determination and Statistical Analysis

Analytical parameters were determined as previously described [45,46,47]. Briefly, the analytical specificity (inclusivity/exclusivity) of the RT-qPCR assays were evaluated in singleplex and multiplex using nucleic acids extracted from the panel of prototype viruses associated with enteric, respiratory, and systemic diseases in dogs and cats, as well as different SARS-CoV-2 variants of concern (VOC). Standard curves were generated using a ten-fold dilution of *IVT* RNA (10^7^ to 10^2^ copies/μL) in triplicate. The analytical sensitivity was assessed by calculating the following parameters: coefficient of determination (R2), amplification efficiency (E [%]), Limit of detection with 95% confidence (LOD_95%_) and cycle threshold (Ct) cut-off values. R^2^ was calculated to assess curve fitness. E [%] was calculated after regression analysis using the following formula: E = [10^–1/slope^ − 1] × 100. LOD_95%_ of each assay was determined by statistical probit analysis (non-linear regression model) using SPSS 14.0 software (SPSS Inc., Chicago, IL, USA) from twelve replicates per dilution ranging from 10^3^ to 10^0^ copies/μL. Ct cut-off values were determined using the following formula: Ct cut-off = Average replicate values of the endpoint dilution + (3 × standard deviation [SD]) [53]. Intra-run variability (repeatability) and inter-run variability (reproducibility) were determined by performing 12 replicates on the same run or three replicates on two independent runs of *IVT* RNA containing 10^5^ to 10^3^ copies/μL, respectively. The coefficient of variation (%CV) was calculated using the following formula: %CV = 100 × (standard deviation of replicates [log_10_ copies/µL] ÷ average of replicates [log_10_ copies/µL]). All graphs were created using GraphPad Prism v9.3.1 statistical analysis software (GraphPad, San Diego, CA, USA).

## 3. Results

### 3.1. Analytical Specificity of the Singleplex and Multiplex Assays for the Detection of Canine and Feline Enteric Viruses

The analytical specificity (inclusivity/exclusivity) of the seven RT-qPCR assays were evaluated in singleplex and multiplex using a panel of prototype viruses associated with enteric, respiratory, and systemic diseases in dogs and cats, as well as different SARS-CoV-2 variants of concern (VOC). All assays used and developed in this study showed exclusive specificity for their respective targets and did not cross-react between each other under multiplex conditions (Supplementary Figure S2).

### 3.2. Analytical Sensitivity of the Singleplex and Multiplex Assays for the Detection of Canine and Feline Enteric Viruses

**Figure 1:**
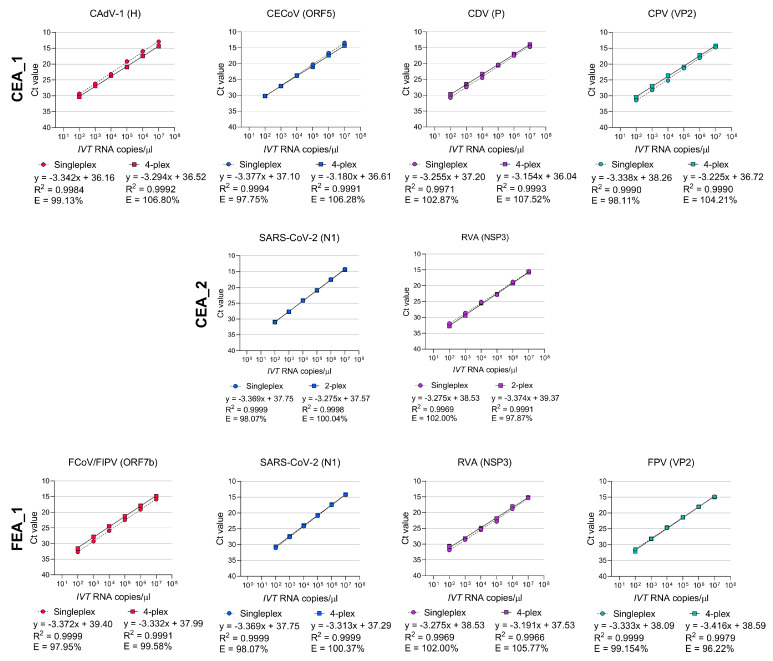
Comparison of analytical sensitivity of each singleplex and multiplex RT-qPCR assays for the detection of SARS-CoV-2 and canine and feline enteric viruses. CEA: Canine enteric assay; Ct: Cycle threshold; FEA: Feline enteric assay; *IVT* RNA: in vitro transcribed RNA; R^2^: linearity; E: Efficiency.

To evaluate the analytical sensitivity of all assays in singleplex and multiplex format, a linear standard curve was generated by performing ten-fold serial dilutions (10^7^ copies/µL to 10^2^ copies/µL) of *IVT* RNA containing the target sequences (Figure 1).

Whenever these assays were used in singleplex and in multiplex, the coefficient of linear regression (R^2^) was ≥ 0.997 and the amplification efficiency was between 96.22% and 107.52% (Table 3), indicating excellent amplification. The lower limit of detection (LOD_95%_) varied between <1 and 25 genome copies/µL for each assay in singleplex format and varied between 5 and 35 genome copies/µL for each assay in multiplex format (Table 3, Figure S3). A similar detection rate limit (100%) was calculated for each assay when used in both singleplex and multiplex conditions (1 to 100 copies/µL) and the Ct cut-off values ranged from 35 to 40 (Table 3). Altogether, these results demonstrate the high analytical sensitivity of our panel of RT-qPCR assays for the detection of canine and feline enteric viruses. In addition, no impact on the analytical sensitivity was observed when these assays were used under multiplex conditions.

### 3.3. Repeatability and Reproducibility of the Multiplex TaqMan^®^ RT-qPCR Assays for the Detection of Canine and Feline Enteric Viruses

The repeatability and reproducibility of the developed multiplex assays were measured by determining the intra-run and inter-run imprecision, respectively. A range of three concentrations—10^5^ copies/µL (high target concentration), 10^4^ copies/µL (medium target concentration), and 10^3^ copies/µL (low target concentration)—of *IVT* RNA was used to determine the coefficient of variability (CV) of each assay. Both intra- and inter-run imprecision were <4% for the high, medium, and low concentration (Table 4). These data indicate that all multiplex assays have a high repeatability and reproducibility.

### 3.4. Assays Validation on Clinical Samples Collected from Dogs with Clinical Signs of Enteric Disease

Both CEA_1 and CEA_2 multiplex assays were used to analyze 27 clinical samples collected from domestic dogs with clinical signs of enteric disease. Eight (29.6%) samples were positive for one of the seven viruses tested: four (14.8%) were positive for CECoV, two (7.4%) were positive for CDV, one (3.7%) was positive for CPV, and one (3.7%) was positive for SARS-CoV-2 (Table 5). The SARS-CoV-2 positive rectal swab was collected from a household case of COVID-19 in 2021. The dog was a 4-year-old female goldendoodle presenting with leukocytosis, fever, lethargy, anorexia, and semi-solid stools for 48 h prior to sample collection. CAdV-1 and RVA, as well as co-infections, were not detected in the tested samples.

### 3.5. Assays Validation on Clinical Samples Collected from Cats with Clinical Signs of Enteric Disease

The FEA_1 multiplex assay was used to analyze 31 clinical samples collected from domestic and feral cats. Among these samples, eleven (35.5%) tested positive for one of the four viruses tested: six (19.4%) were positive for FPV, four (12.9%) were positive for FcoV, one (3.2%) was positive for SARS-CoV-2, and none of the samples were positive for RVA (Table 6). The SARS-CoV-2-positive sample was collected in 2021 from the feces of a 6-year-old captive male African lion unvaccinated against SARS-CoV-2, who presented coughing and mild lethargy. The National Veterinary Services Laboratories in Ames, IA, conducted partial sequencing of the SARS-CoV-2 Spike protein gene (ORF2), revealing that the SARS-CoV-2-positive sample corresponded to the Delta variant (clade B.1.617.2). Co-infection was not detected in any of the tested samples. 

## 4. Discussion

Gastrointestinal infections in dogs and cats can be caused by a wide range of pathogens, including viruses, bacteria, and parasites. Among the viral agents, coronaviruses such as CECoV and FCoV/FIPV are well-known to be responsible for inducing gastroenteric disorders in canine and feline populations [54,55]. Additionally, SARS-CoV-2, the causative agent of COVID-19, has been reported to infect dogs and cats, and can be found in fecal samples collected from naturally and experimentally infected companion animals [15,16,17,18,19,20,21,23]. Parvoviruses (CPV and FPV) are also well known to induce severe gastroenteric disease and are often fatal for puppies and kittens [36,56]. Co-infection and recombination between CPV and FPV have also been documented [57,58]. CAdV-1 and CDV are capable of inducing a wide range of clinical signs in dogs, as they are considered multisystemic infections [59,60]. While RVA is a significant cause of diarrhea in children and various animal species [42,61,62,63], canine and feline RVA typically do not lead to severe illness and are not considered a major gastrointestinal pathogen in these species. However, similarities in their genomic constellation with some human RVA strains indicate that the latter have likely originated from canine and feline RVA [64,65]. Detecting canine and feline RVA is crucial not only for monitoring animal health but also due to its zoonotic potential. Therefore, its detection is essential to prevent potential transmission of the virus from animals to humans.

Rapid diagnosis of the causative agent in gastrointestinal disease is crucial to provide the appropriate treatment and implement vaccination and control measures. However, confirmatory diagnosis requires laboratory testing. In this study, a multiplex one-step RT-qPCR panel was developed and validated for the detection of SARS-CoV-2 and the most common canine and feline enteric viruses. These multiplex assays were designed using a combination of well-established qPCR/RT-qPCR singleplex assays [42,48,49,50,51,52,66]. The detection of canine and feline RVA was included in our panel by using the assay previously established by Freman et al. [48], which is now commonly used for the detection of equine RVA [42,47]. Addition of a nucleotide degeneracy in position 2 of the NVP3-FDeg primer (C→Y) was added. This assay was able to detect the canine RVA prototype without detection of other canine and feline viruses. The specificity of this assay was not assessed against feline RVA, as no prototype strain is currently available. However, in silico analysis demonstrated high specificity of this assay against the few canine and feline NSP3 RVA nucleotide sequences available, with only 1.4% of nucleotide differences in seven of the 26 available sequences (Figure S1).

In this study, analytical performance was appropriate under multiplex conditions, with a limit of detection (LOD_95_) ranging from 5 to 35 genome copies/µL. Even though under singleplex conditions the LOD_95_ was ranged between <1 to 25 genome copies/µL, this difference compared to multiplex conditions is considered negligible as it is a <10-fold difference and might be attributed to competition between assays and potential reagent exhaustion, as previously suggested [47]. The multiplex assays demonstrated excellent reproducibility and repeatability, indicating their high accuracy. The analytical parameters of these multiplex assays were similar to other assays recently developed in our laboratory for the detection of canine and feline respiratory pathogens [45,46]. Moreover, in this study, the LOD_95_ for CPV and CECoV showed a one log_10_ improvement compared to previously published multiplex qPCR assays (LOD_95_ was 1 × 10^2^ copies/μL) [44], and a three log_10_ improvement compared to a multiplex RT-PCR assay designed for detecting CPV, CECoV, and CAdV-1 [67]. Similarly, our FEA_1 assay displayed a reduction of more than one log_10_ in detecting FPV when compared to another multiplex RT-qPCR assay designed for detecting FPV, feline bocavirus 1, feline astrovirus, and feline kobuvirus [68]. Furthermore, all the previous assays were conducted in two separate steps (reverse-transcription and subsequent qPCR amplification), while our newly developed one-step assays combined both the reverse-transcription and qPCR amplification steps. This significantly reduces labor, minimizes the risk of pipetting errors, and shortens the time required to obtain results. To our knowledge, no previous multiplex RT-qPCR assays were developed for the simultaneous detection of the six canine enteric viruses (CPV, CECoV, CAdV-1, CDV (CEA_1), RVA, and SARS-CoV (CEA_2)); and for the detection of the four feline enteric viruses (FCoV/FIPV, FPV, RVA, and SARS-CoV-2 (FEA_1)). The sensitivity of the qPCR reaction may vary depending on the reagents and thermocycler used. The parameters established in this study were derived from a specific platform. Consequently, any modification of reagents or thermocycler would necessitate a reassessment of the analytical sensitivity.

As only 29.6% and 35.5% of the samples collected from dogs and cats suspected to have gastroenteric disease tested positive for the enteric viruses examined in this study, it is presumed that other pathogens, such as bacteria and parasites, may be involved as primary causative agents. No RVA was detected in the collected specimens. However, it is important to note that, due to the limited number of samples collected and tested in this study, the absence of canine and feline RVA detection does not necessarily indicate their absence in this particular region of the world. To determine the prevalence and genetic diversity of canine and feline RVA strains circulating in the USA, a larger-scale study is necessary. Interestingly, SARS-CoV-2 was detected in a rectal swab collected from a household dog and feces from a captive feral cat. This underscores the importance of detecting such viruses to prevent potential transmission at the human–animal interface.

The main limitation of this study is associated with the limited number of available samples during the study period and, therefore, limited our ability to assess its clinical performance. In addition, the absence of coinfection is most likely due to the limited number of samples collected during this study. The versatility of this panel allows the possibility of expansion to include additional viral targets or the incorporation of emerging viruses in the future.

## 5. Conclusions

In conclusion, this article describes the development and validation of a panel of robust multiplex RT-qPCR assays (CEA_1, CEA_2, and FEA_1) capable of simultaneous detection of SARS-CoV-2 and the most relevant enteric viruses in dogs and cats. The implementation of such a comprehensive diagnostic tool has the potential to improve disease surveillance, diagnosis, and management of potential zoonotic diseases. 

## Figures and Tables

**Table 1:** Panel of prototype SARS-CoV-2 variants of concern, canine and feline enteric viruses, and canine and feline respiratory viruses used to assess the specificity of each RT-qPCR assay.

Pathogens	Reference Strain	Source
SARS-CoV-2 USA-WA1/2020	NR-52281	BEI Resources
SARS-CoV-2 Alpha (B.1.1.7)	NR-54020	BEI Resources
SARS-CoV-2 Beta (B.1.351)	NR-55282	BEI Resources
SARS-CoV-2 Delta (B.1.617.2)	NR-55671	BEI Resources
SARS-CoV-2 Omicron (B.1.1.529)	NR-56461	BEI Resources
Canine Distemper Virus (CDV) Lederle Avirulent	NR-3845	BEI Resources
Canine Parvovirus (CPV)	VR-953^TM^	ATCC^®^
Canine Enteric Coronavirus (CECoV), UCD1	NR-868	BEI Resources
Canine Adenovirus 1 (CAdV-1)	VR-293^TM^	ATCC^®^
Canine Rotavirus (CRV)	VR-964 ^TM^	ATCC^®^
Feline Panleukopenia Virus (FPV)	VR-648^TM^	ATCC^®^
Feline Coronavirus (FCoV)	L1911562	LADDL
Feline Infectious Peritonitis Virus (FIPV)	NR-43287	BEI Resources
Canine Adenovirus 2 (CAdV-2)	VR-800^TM^	ATCC^®^
Canine Respiratory Coronavirus (CRCoV)	VSL-1471	Cornell University ^a^
Canine Parainfluenza Virus (CPiV)	VR-399^TM^	ATCC^®^
Canine Herpesvirus 1 (CHV-1)	VR-522^TM^	ATCC^®^
Canine Pneumovirus (CPnV)	124423	LADDL
Canine Influenza A H3N2	VLS-1355	Cornell University ^a^
Canine Influenza A H3N8	A/Ca/FL/15592/04	Cornell University ^b^
Felid Herpesvirus 1 (FHV-1)	VR-814^TM^	ATCC^®^
Feline Calicivirus (FCV)	VR-782^TM^	ATCC^®^

^a^ Kindly provided by Dr. Diego Diel. ^b^ Kindly provided by Dr. Edward Dubovi.

**Table 2:** Primers and probe sequences used for the detection of SARS-CoV-2 and other viruses associated with enteric diseases in dogs and cats.

Target (Gene)	Oligonucleotide ID	Primers and Probe Sequences (5′-3′)	Nucleotide Position	Product Size (bp)	GenBank Accession #	Reference
SARS-CoV-2 (Nucleocapsid)	2019-nCoV_N1-F	GACCCCAAAATCAGCGAAAT	28,287–28,306	72	MN985325.1	[42]
	2019-nCoV_N1-R	TCTGGTTACTGCCAGTTGAATCTG	28,358–28,335			
	2019-nCoV_N1-P	FAM-ACCCCGCATTACGTTTGGTGGACC-QSY	28,309–28,332			
CDV (Phosphoprotein)	CDV_P-F	ACTATTGAGAGACCTCCAGCTGAAA	1296–1320	79	AB028914.1	[45]
	CDV_P-R	TGCGGTATCCTTCGGTTTGT	1374–1355			
	CDV_P-P	JUN-CCGATTGCCGAGCTAGACTCTTTGTCA-QSY	1352–1326			
CPV/FPV (VP2)	CPV-For	AAACAGGAATTAACTATACTAATATATTTA	4102–4131	93	M38245.1	[49]
	CPV-Rev	AAATTTGACCATTTGGATAAACT	4194–4172			
	CPV-Pb	VIC-TGGTCCTTTAACTGCATTAAATAATGTACC-QSY	4139–4168			
CECoV (ORF5)	CcoV-For	TTGATCGTTTTTATAACGGTTCTACAA	26,449–26,475	99	JQ404409.1	[50]
	CcoV-Rev	AATGGGCCATAATAGCCACATAAT	26,547–26,524			
	CcoV-Pb	FAM-ACCTCAATTTAGCTGGTTCGTGTATGGCATT-QSY	26,484–26,514			
CAdV-1 (Hexon)	CAV-F	AGTAATGGAAACCTAGGGG	17,681–17,699 17,760–17,743 17,710–17,734	80	U55001.1	[51]
	CAV-R	TCTGTGTTTCTGTCTTGC				
	CAV1-Pb	ABY-TCAATCGTCTCAACTAAATGCCGTG-QSY				
RVA (NSP3)	NVP3-FDeg	AYCATCTWCACRTGACCCTC	992–1011	87	MT364832.1	[42,48] with modification
	NVP3-R1	GGTCACATAACGCCCCTATA	1078–1059			
	NVP3-Probe	JUN-ATGAGCACAATAGTTAAAAGCTAACACTGTCAA-QSY	1013–1045			
FCoV/FIPV (ORF7b)	FCoV1128f	GATTTGATTTGGCAATGCTAGATTT	29,150–29,170	102	FJ938059.1	[52]
	FCoV1229r	AACAATCACTAGATCCAGACGTTAGCT	29,251–29,225			
	FCoV1200p	ABY-TCCATTGTTGGCTCGTCATAGCGGA-QSY	29,198–29,222			

ABY, ABY^TM^ dye; F: forward primer; FAM, 6-carboxyfluorescein dye; JUN, JUN™ dye; P: probe; QSY, QSY™ quencher; R: reverse primer; VIC, VIC™ dye.

**Table 3:** Analytical performance of singleplex and multiplex RT-qPCR assays for the detection of SARS-CoV-2 and canine and feline enteric viruses.

Panel	Target	Parameter	Slope	Linearity (R^2^)	Efficiency (E [%])	LOD_95%_ (Copies/µL)	Detection Rate Limit (Copies/µL)	Ct Cut-Off
CEA_1	CAdV-1 (H)	Singleplex	−3.343	0.9984	99.13	25	100	31
		4-plex	−3.169	0.9992	106.80	6	10	38
	CECoV (ORF5)	Singleplex	−3.377	0.9994	97.75	4	10	36
		4-plex	−3.180	0.9991	106.28	6	10	40
	CDV (P)	Singleplex	−3.255	0.9971	102.87	4	10	36
		4-plex	−3.154	0.9993	107.52	5	10	38
	CPV (VP2)	Singleplex	−3.368	0.9990	98.11	4	10	36
		4-plex	−3.225	0.9990	104.21	14	100	32
CEA_2	SARS-CoV-2 (N1)	Singleplex	−3.369	0.9999	98.07	<1	10	40
		2-plex	−3.321	0.9998	100.04	5	10	35
	RVA (NSP3)	Singleplex	−3.275	0.9969	102.00	14	100	34
		2-plex	−3.374	0.9991	97.87	14	100	33
FEA_1	FCoV (ORF7b)	Singleplex	−3.372	0.9999	97.95	11	100	35
		4-plex	−3.332	0.9991	99.58	14	100	38
	SARS-CoV-2 (N1)	Singleplex	−3.369	0.9999	98.07	<1	1	40
		4-plex	−3.313	0.9999	100.37	6	10	35
	RVA (NSP3)	Singleplex	−3.275	0.9969	102.00	14	100	34
		4-plex	−3.191	0.9966	105.77	35	100	33
	FPV (VP2)	Singleplex	−3.333	0.9999	99.54	<1	1	40
		4-plex	−3.416	0.9979	96.22	8	10	39

CEA: Canine enteric assay; FEA: Feline enteric assay; R^2^: Linearity; LOD_95%_: Limit of detection 95%; Ct: Cycle threshold.

**Table 4:** Precision assessment of each multiplex RT-qPCR assay.

Panel	Target	Intra-Run Variability CV (%) ^#^			Inter-Run Variability CV (%) ^#^		
		10^5^ Copies/µL	10^4^ Copies/µL	10^3^ Copies/µL	10^5^ Copies/µL	10^4^ Copies/µL	10^3^ Copies/µL
CEA_1	CAdV-1 (H)	0.93	1.40	2.61	1.11	1.16	1.84
	CECoV (ORF5)	0.73	0.99	2.88	0.89	2.66	3.57
	CDV (P)	1.54	2.12	3.84	0.75	1.36	2.65
	CPV (VP2)	1.17	1.15	3.14	0.83	0.83	2.48
CEA_2	SARS-CoV-2 (N1)	1.38	2.67	2.59	0.45	1.13	1.93
	RVA (NSP3)	2.38	1.00	1.45	1.16	2.61	3.05
FEA_1	FCoV (ORF7b)	0.71	1.34	1.17	0.79	1.15	1.90
	SARS-CoV-2 (N1)	0.57	0.79	1.20	0.81	1.78	2.05
	RVA (NSP3)	3.55	3.62	2.71	2.80	2.44	2.99
	FPV (VP2)	0.90	0.94	0.87	1.53	0.39	0.30

^#^ CV (%): Coefficient of variation = (standard deviation of replicates [log_10_ copies/µL] ÷ Average of replicates [log_10_ copies/µL]) × 100.

**Table 5:** Detection rate of canine enteric-associated viruses in the clinical specimens used to evaluate the CEA_1 and CEA_2 assays.

Viruses	No. of Positive (*n* = 8/27)	Positive (%) (29.6%)
CDV	2	7.4
CPV	1	3.7
CECoV	4	14.8
CadV-1	0	0
RVA	0	0
SARS-CoV-2	1	3.7

**Table 6:** Detection rate of feline enteric-associated viruses in the clinical specimens used to evaluate the FEA_1 assay.

Viruses	Positive (*n* = 11/31)	Positive (%) (35.5%)
FCoV	4	12.9
FPV	6	19.4
RVA	0	0
SARS-CoV-2	1	3.2

## Data Availability

The data presented in this study are available on request from the corresponding author.

## Supplementary Materials

The following supporting information can be downloaded at: https://www.mdpi.com/article/10.3390/v15091890/s1, Figure S1: Canine and feline RVA-NSP3 nucleotide sequences alignment with the NVP3-FDeg primer, the NVP3-R1 primer and NVP3-Probe; Figure S2: Assessment of the specificity of each RT-qPCR assay using nucleic acids derived from prototype viruses; Figure S3: Limit of detection with 95% confidence (LOD_95%_) determination of singleplex and multiplex RT-qPCR assays.
