# Supplementary material for: Multiplex One-Step RT-qPCR Assays for Simultaneous Detection of SARS-CoV-2 and Other Enteric Viruses of Dogs and Cats
Source: Viruses. 2023 Sep 7;15(9):1890. doi: 10.3390/v15091890 (PMC10534472; doi:10.3390/v15091890)
Supplement: Supplementary file 1 [file viruses-15-01890-s001.zip › viruses-2579313-supplementary.pdf]

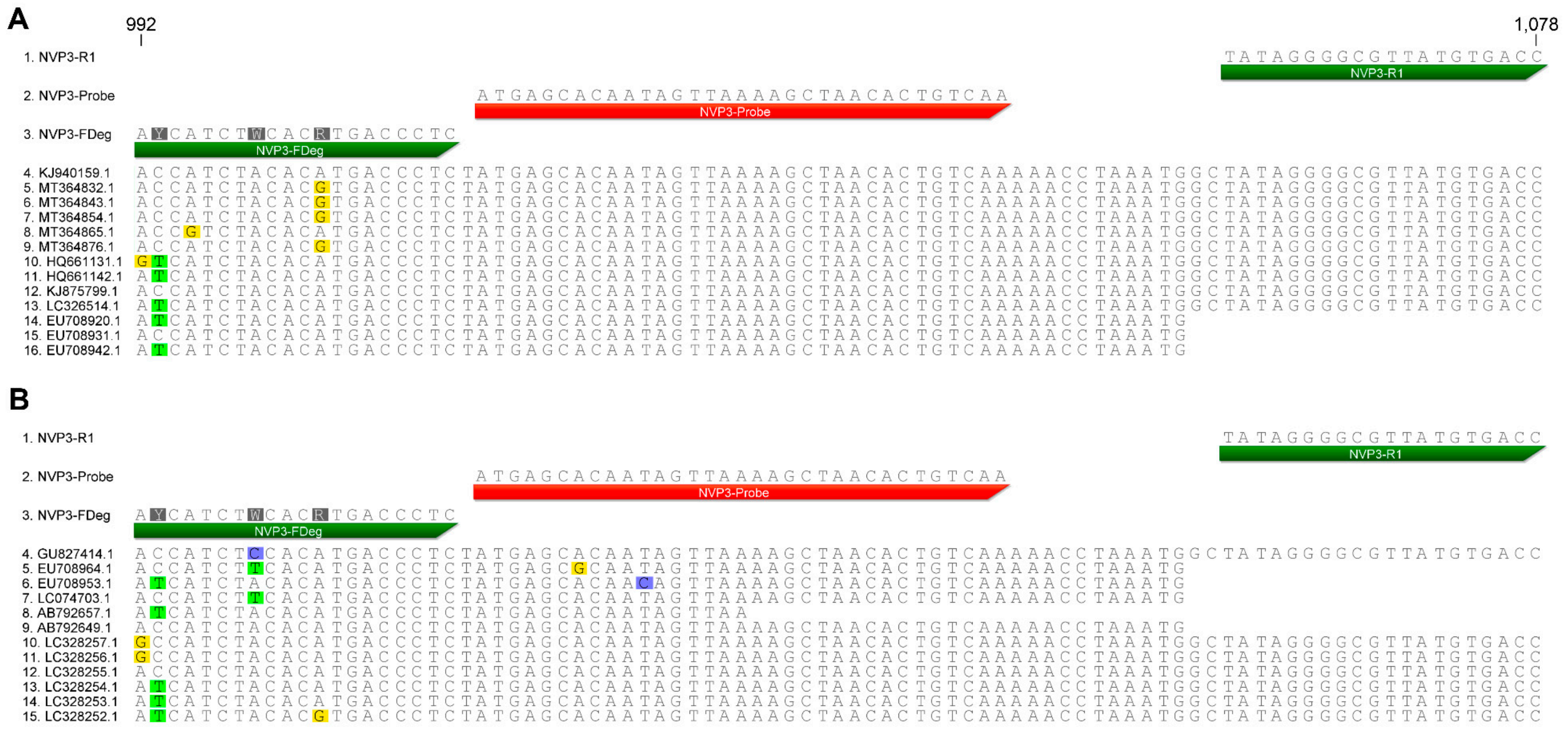

Figure S1. Canine and feline RVA-NSP3 nucleotide sequence alignment with the NVP3-FDeg primer, the NVP3-R1 primer and NVP3-Probe. A) Nucleotide sequence alignment between canine RVA NSP3 gene sequences downloaded from GenBank (n=13) and the primers and probe used for the detection of RVA. **B**) Nucleotide sequence alignment between feline RVA NSP3 gene sequences downloaded from Genbank (n=13) and the primers and probe used for the detection of RVA. Nucleotide differences between the field strain and the primers and probes are boxed and in colors. No nucleotide differences were noted between 11 and 7 canine and feline field strains sequences, respectively. One nucleotide difference (1.4%) was noted in the nucleotide sequence of 2 and 5 canine and feline field strains, respectively, with the primers and probes used. R = A or G; W = A or T; Y = C or T.

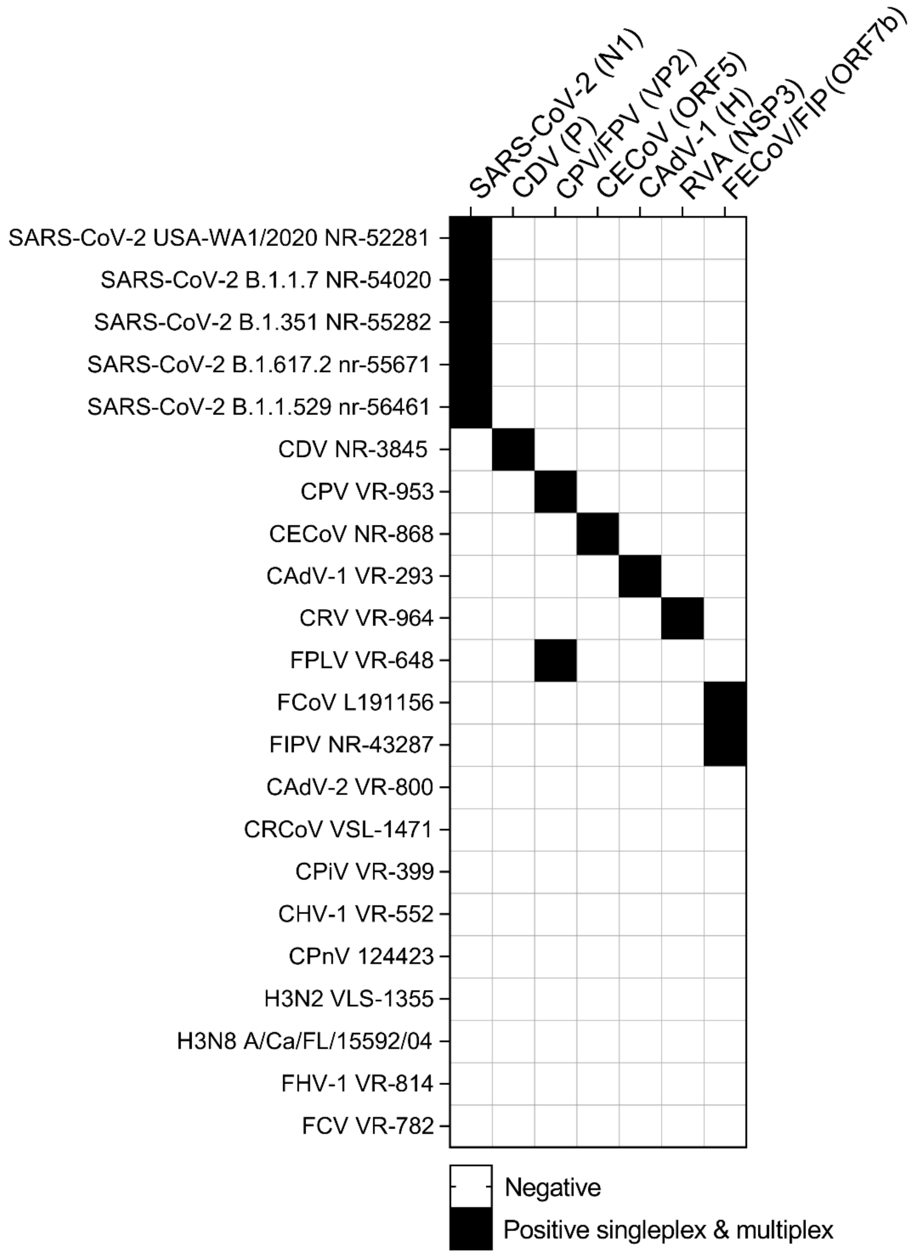

Figure S2: Assessment of the specificity of each RT-qPCR assay using nucleic acids derived from prototype viruses. Each column corresponds to one specific RT-qPCR assay and each row corresponds to one specific reference virus strain. Specificity was assessed for each assay in singleplex and in multiplex formats. White cases correspond to the absence of detection while black cases correspond to DNA/RNA amplification in both singleplex and multiplex assays.

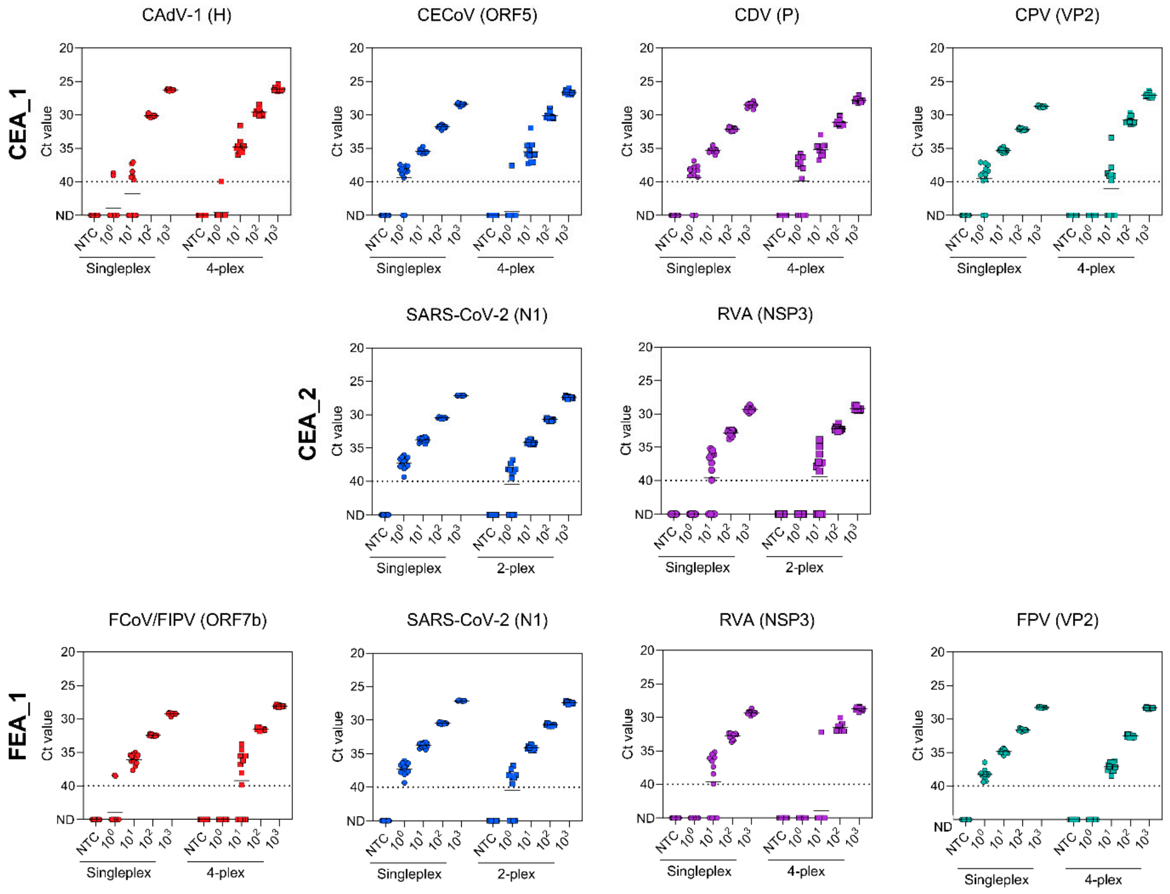

Figure S3: Limit of detection with 95% confidence (LOD95%) determination of singleplex and multiplex RT-qPCR assays. Each assay was performed using 12 replicates ranging from 10^3 to 10^0 copies/µl of *IVT* RNA. Each circle and square indicate the Ct value of one replicate obtained by singleplex and multiplex amplification, respectively. Short solid lines indicate the median Ct value and dashed lines indicate the detection limit. ND: not detected; NTC: no template control.
